# Regulation of thymus-dependent and thymus-independent production of immunoglobulin G subclasses by Galpha_12 _and Galpha_13_

**DOI:** 10.1186/1750-2187-3-12

**Published:** 2008-07-12

**Authors:** Song Jin Lee, Woo Hyung Lee, Chang Ho Lee, Sang Geon Kim

**Affiliations:** 1 College of Pharmacy and Research Institute of Pharmaceutical Sciences, Seoul National University, Seoul 151-742, Korea; 2 Yuhan Corp., Daebang-dong, Dongjak-gu, Seoul 156-754, Korea; 3 Department of Pharmacology and Institute of Biomedical Science, College of Medicine, Hanyang University, Seoul 133-791, Korea

## Abstract

**Background:**

A previous study from this laboratory showed that Gα_12 _members participate in the production of inflammatory cytokines. In spite of the identification of B cell homeostasis responses regulated by Gα_13_, the functional roles of Gα_12 _members in the production of immunoglobulin (Ig) isotypes remained unknown. This study investigated whether Gα_12 _members are involved in the Ig isotype antibody production with the purpose of establishing their functions in thymus-dependent and thymus-independent humoral responses.

**Results:**

Mice lacking Gα_12 _and/or Gα_13 _showed an impaired antigen-specific antibody production promoted by challenge(s) of ovalbumin or trinitrophenyl-lipopolysaccharide (TNP-LPS), used for thymus-dependent and thymus-independent stimuli, respectively. Homozygous knockout (KO) of Gα_12 _or double heterozygous KO of Gα_12_/Gα_13 _significantly reduced the antigen-specific total IgG level after multiple ovalbumin immunizations with decreases in the production of IgG1, IgG2a and IgG2b subclasses, as compared to wild type control. In contrast, IgM production was not decreased. Moreover, mice deficient in Gα_12 _or partially deficient in Gα_13 _or Gα_12_/Gα_13 _showed significantly low production of IgG2b in response to TNP-LPS. In TNP-LPS-injected mice, IgG1 and IgG2a productions were unaffected by the G protein KOs.

**Conclusion:**

Our results demonstrate that both Gα_12 _and Gα_13 _are essentially involved in thymus-dependent and independent production of IgG subclasses, implying that the G-proteins contribute to the process of antigen-specific IgG antibody production.

## Background

G-protein-coupled receptors (GPCR) regulate signal transduction in cells, participating in a variety of biological processes [1]. An activated GPCR makes a complex with a G-protein defined by α subunit. Gα subunits consist with Gα_s_, Gα_i/o_, Gα_q _and Gα_12 _[1]. The members of Gα_12 _family consisting of Gα_12 _and Gα_13 _are critical mediators in regulating effectors or cellular responses. Their interactions with specific Rho guanine nucleotide exchanging factor result in small GTPase RhoA activation, leading to diverse biological functions such as migration and maturation of B cells, morphology and motility of cells, and smooth muscle contraction [2-4].

Activated B cells proliferate and differentiate into immunoglobulin (Ig)-producing plasma cells or long-lived memory cells [5]. It is well recognized that Ig antibody (Ab) production by B cells is the critical process in the defense against infection. Immune cells express a variety of GPCRs, whose activations result in coupling of G-proteins for signal amplifications [1]. In particular, it has been shown that Gα_12 _and Gα_13 _regulate the homeostasis of marginal zone B cells, in which the G-proteins activated LSC, a p115RhoGEF, for humoral responses [2]. Our studies showed that activation of Gα_12_and/or Gα_13 _leads to the induction of iNOS and COX-2 through NF-κB, an essential transcription factor required for immune responses [6,7]. Moreover, sphingosine 1-phospate (S1P), a representative lysophospholipid GPCR ligand in blood or lymph nodes, disseminates the signal to B cells via S1P_3 _GPCR to position marginal zone B cells [8].

Despite the identified role of Gα_12_/Gα_13 _in the regulation of mariginal zone B cell biology [2], the functions of Gα_12_/Gα_13 _in Ig production have not been completely identified. In view of complex network of the immune system and involvements of diverse cell types, we investigated whether the Gα_12 _family members regulate the production of Ig classes and IgG subclasses with particular emphasis on their roles in thymus-dependent or thymus-independent immunity. In this study, Ab production was measured after challenges of two different types of antigens. To elicit thymus-dependent immune response, wild type (WT) or Gα_12 _and/or Gα_13 _heterozygous or homozygous knockout (KO) mice were immunized with ovalbumin (OVA). In another set, the animals were injected with trinitrophenyl-lipopolysaccharide (TNP-LPS) to promote thymus-independent Ig production. Through these *in vivo *analyses, it was found that Gα_12 _and Gα_13 _have regulatory functions in producing IgG subclasses. This information may bring insight in understanding the role of Gα_12 _members in humoral immune responses.

## Results

**Figure 1:**
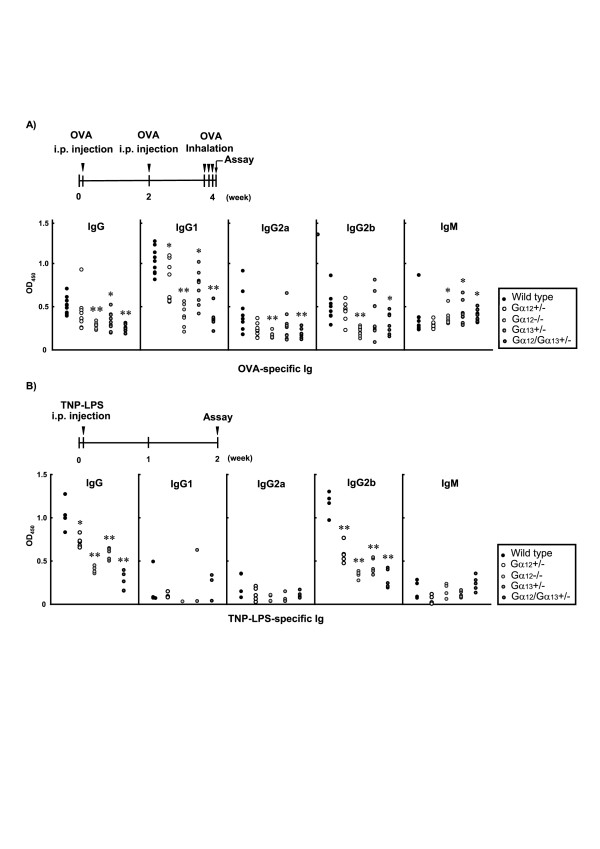
**Antigen-specific Ig production in mice immunized with antigens.** A) OVA-specific Ig production in mice immunized with OVA. Wild type (WT) or Gα_12_/Gα_13 _knockout (KO) mice were i.p injected with OVA on day 1 and day 14, respectively, and challenged by nebulizing 1% OVA solution for 3 consecutive days (days 26–28). Secondary immune responses for OVA-specific total IgG or IgM class, or OVA-specific IgG subclasses in sera were assessed on day 29. B) Ig production in mice injected with a single dose of TNP-LPS. Wild type (WT) or Gα_12_/Gα_13 _knockout (KO) mice were injected with TNP-LPS (25 μg/mouse) on day 1. After 2 weeks, TNP-LPS-specific total IgG or IgM class, or TNP-LPS-specific IgG subclasses were assayed in the sera. Preimmune sera obtained on day 0 were used as controls. Number of animals in each treatment group = 10. Data represent means ± S.E.M. (significant compared to the respective Ig in WT mice *p < 0.05, **p < 0.01). OVA, ovalbumin; TNP, trinitrophenyl; LPS, lipopolysaccharide.

To determine the possible role(s) of Gα_12 _and/or Gα_13 _in the regulation of thymus-dependent Ab production, the Ig titers were measured in WT mice or mice deficient in Gα_12_/Gα_13 _that had been immunized with OVA and booster-injected with the same antigen 2 weeks after, which was followed by OVA nebulizations (Fig. 1A). After the immunizations and nebulizations, WT mice showed normal OVA-specific IgG and IgM production (Fig. 1A, Table 1). In contrast, homozygous absence of the *Gα*_12 _gene significantly impaired OVA-specific IgG production. When OVA-specific Ig subclass contents were assessed, Gα_12 _deficiency decreased the production of IgG1, IgG2a and IgG2b subclasses compared to those in WT. Also, double heterozygous deletions of Gα_12 _and Gα_13 _significantly reduced OVA-specific IgG content with decreases in the levels of IgG1, IgG2a or IgG2b subclasses. The increased production of IgM was unaffected by the absence of Gα_12 _and/or Gα_13_. In this assay, we could not use the animals with homozygous *Gα*_13 _KO because these mice die before birth [9]. Our data demonstrated that Gα_12 _and Gα_13 _are both required for thymus-dependent IgG1, IgG2a and IgG2b production.

**Table 1:** Production of Ig classes and IgG subclasses in WT mice or mice deficient in Gα_12 _and/or Gα_13_

	**Thymus-dependent Ig production**					**Thymus-independent Ig production**				
		
	WT	Gα_12 _+/-	Gα_12 _-/-	Gα_13 _+/-	Gα_12_/Gα_13_+/-	WT	Gα_12 _+/-	Gα_12 _-/-	Gα_13 _+/-	Gα_12_/Gα_13 _+/-
IgG	++	++	+	+	+	++++	+++	+	++	+
IgG1	+++	+++	+	++	+	+	+	+	+	+
IgG2a	++	+	+	+	+	+	+	+	+	+
IgG2b	++	++	+	+	+	++++	+++	+	++	+
IgG3	t	t	t	t	t	+	+	+	+	++
IgM	+	+	+	++	+	+	+	+	+	+
IgA	t	t	t	t	t	t	t	t	t	t
IgE	t	t	t	t	t	Not detected				

The relative Ig production was defined as followings: -, OD_450 _< 0.15; +, OD_450 _0.15–0.4; ++, OD_450 _0.4–0.75; +++, OD_450 _0.75–1.0; and ++++, OD_450 _> 1.0. The absorbance at 450 nm (OD_450_) was measured using ELISA assays. Number of animals in each treatment group = 10

LPS is a classic mitogenic stimulus that activates the innate B cell receptors [5]. It is known that LPS stimulation activates B cells without T cell help. In another set of experiments, we determined the roles of Gα_12_/Gα_13 _for the regulation of B cell immune response stimulated by TNP-LPS, a well-known thymus-independent antigen. In WT mice, total Ig production by TNP-LPS was greatly promoted 2 weeks after (Fig. 1B). The heterozygous or homozygous KO of Gα_12 _inhibited TNP-LPS-specific IgG production in a gene dose-dependent manner. In the mice partially deficient in Gα_13 _or both Gα_12 _and Gα_13_, the degrees of TNP-LPS-specific IgG production were also decreased compared to WT mice challenged with the same antigen (Fig. 1B, Table 1). We found that decreases in TNP-LPS-specific IgG titer by the lack(s) of Gα_12 _and/or both Gα_12 _and Gα_13 _exactly matched with decreases in TNP-LPS-specific IgG2b, indicating the specific role of Gα_12 _and Gα_13 _for the production of IgG2b subclass. TNP-LPS-specific IgG1, IgG2a, and IgM contents were not changed by the gene KOs, suggesting that the change in TNP-LPS-specific IgG production might be due to that in IgG2b. Thus, Ig production in response to TNP-LPS was found to be regulated by Gα_12 _and Gα_13_. All of these results provide evidence that Gα_12 _and Gα_13 _are both required for regulating thymus-dependent and thymus-independent production of IgG subclasses.

## Discussion

There are two distinctive pathways that induce B cell responses (that is, thymus-dependent and thymus-independent B cell activation) [10,11]. In the present study, we demonstrated for the first time that Gα_12 _and Gα_13 _are both required for thymus-dependent production of IgG subclasses. Significant changes in the humoral response, particularly in regulating IgG, by the lack(s) of Gα_12 _and Gα_13 _highlight the need to consider the G-protein pathway as one of important regulatory controls for T-cell dependent immune network.

Affinity maturation of B cells, which need Th1 cytokines, consists with the processes of clonal proliferation, somatic hypermutation, and selection [10]. Th1 cytokines such as interferon-γ (IFN-γ) stimulate IgG2a, IgG2b, and IgG3 production [11]. Therefore, our results showing that Gα_12 _and Gα_13 _deficiency notably decreased IgG2a and IgG2b subclasses in an OVA-immunized model suggest that the affinity maturation processes of B cells might be affected by Gα_12 _and Gα_13_. Th2 cytokine (e.g., interleukin-4) particularly induces Ig switching to IgG1 and IgE [12]. Our data indicated that the titer of anti-OVA IgG1, but not anti-OVA IgE, was lowered by Gα_12_/Gα_13 _deficiency, which suggested that maturation of B cell to IgG1-producing plasma cell might need the G-protein-mediated signaling processes presumably upon stimulation of Th2 cytokine(s). Collectively, the essential regulatory function of Gα_12_/Gα_13 _in producing IgG subclasses lends support to the hypothesis that helper T cell-dependent B cell activation by antigen requires the G-protein-mediated signaling pathway.

Another type of B cell activation is thymus-independent, which is triggered by viral particles, common bacterial antigens, and TLR ligands without T cell help [5]. B cell activation by thymus-independent antigens (e.g., LPS) causes the production of polyclonal antibody [13]. In our animal model, TNP-LPS-inducible production of IgG2b, but not IgG3, was decreased by the absence of Gα_12 _and Gα_13_, showing that the G-proteins might regulate thymus-independent IgG production by B cells. Because transforming growth factor-β stimulates IgG2b and IgA production [14], the decrease in IgG2b in TNP-LPS-injected mice deficient in Gα_12_/Gα_13 _might be associated with repression of transforming growth factor-β. This possibility is strengthened by our finding that Gα_12_/Gα_13 _regulate transforming growth factor-β production in the liver of mice challenged with dimethylnitrosamine (Lee et al, unpublished data).

Gα_12 _regulates NF-κB-mediated COX-2 induction by S1P in a process mediated by the JNK-dependent ubiquitination and degradation of IκBα [7]. Because antigen-induced cell signaling requires NF-κB and AP-1, decreased activation of the transcription factors may account for altered IgG production in the KO mice. Our results illustrating the role of Gα_12 _and Gα_13 _in IgG production may be explained by other possibilities: that is, (1) the role of Gα_12_/Gα_13 _in B cell reentry into the secondary lymphoid organ and (2) the regulatory role of Gα_12_/Gα_13 _in the specific step of Ab production.

## Methods

### OVA or TNP-LPS Immunizations

All experiments were conducted under the guidelines of the Institutional Animal Use and Care Committee at Seoul National University, Korea. WT and *Gα*_12_*/Gα*_13 _KO mice at the age of 8–10 weeks (25~30 g)(*Gα*_12_+/-, *Gα*_12_-/-, *Gα*_13_+/-, and *Gα*_12_*/Gα*_13_+/-) were used for *in vivo *experiments. To induce OVA-specific Ab production, the mixture of 100 μg endotoxin-free OVA (MP Biomedicals, Aurora, OH) dissolved in PBS and alum (Pierce, IL) was i.p. injected to the mice on day 1. On day 14, the mice were i.p. injected again with 100 μg OVA. The mice were subsequently exposed to aerosol of 1% OVA solution for 30 min once a day on days 26, 27 and 28. In another set of experiments, a single dose of TNP-LPS (Biosearch Technologies, Novato, CA) was i.p. injected to WT and *Gα*_12_*/Gα*_13 _KO mice to assess the production of TNP-LPS-specific Ab. The animals were bled 2 weeks after.

### Enzyme-Linked Immunosorbent Assay (ELISA)

ELISA assays were performed to determine antigen-specific total IgG, IgG1, IgG2a, IgG2b, IgG3, IgA, IgE and IgM in aliquots of diluted serum (1:2000) in a 96-well plate (Maxisorp, Nunc Co., Rochester, NY). Alkaline phosphatase-conjugated goat anti-mouse Ig isotype and IgG subclass (Southern Biotechnology Associates Inc., Birmingham, Ala), and *p*-nitrophenyl phosphate (phosphatase substrate) were used to assay titers.

### Statistical Analysis

One way analysis of variance (ANOVA) procedures were used to assess significant differences among treatment groups. The Newman-Keuls test was used for comparisons of multiple group means.

## Abbreviations

Ab: antibody; GPCRs: G-Protein-coupled receptors; G-proteins: GTP-binding proteins; OVA: ovalbumin; LPS: lipopolysaccharide; TNP: trinitrophenyl; Ig: immunoglobulin; S1P: sphingosine 1-phosphate; WT: wild type; KO: knockout.

## Competing interests

The authors declare that they have no competing interests.

## Authors' contributions

SJL, CHL, SGK designed research; SJL and WHL performed research; SJL and WHL analyzed data; and CHL and SGK wrote the paper. All authors read and approved of the final manuscript.
